# β-synuclein potentiates synaptic vesicle dopamine uptake and rescues dopaminergic neurons from MPTP-induced death in the absence of other synucleins

**DOI:** 10.1016/j.jbc.2021.101375

**Published:** 2021-11-02

**Authors:** Natalia Ninkina, Steven J. Millership, Owen M. Peters, Natalie Connor-Robson, Kirill Chaprov, Arthur T. Kopylov, Alex Montoya, Holger Kramer, Dominic J. Withers, Vladimir L. Buchman

**Affiliations:** 1 School of Biosciences, Cardiff University, Cardiff, United Kingdom; 2 Institute of Physiologically Active Compounds, Russian Academy of Sciences, Chernogolovka, Russian Federation; 3 Metabolic Signalling, MRC London Institute of Medical Sciences, London, United Kingdom; 4 Institute of Clinical Sciences, Faculty of Medicine, Imperial College London, London, United Kingdom; 5 Department of Proteomic Research and Mass Spectrometry, Institute of Biomedical Chemistry, Moscow, Russian Federation

**Keywords:** synuclein, dopaminergic neurons, synapse, vesicles, transgenic mice, dopamine, MPTP toxicity, neurodegenerative disease, Parkinson's disease, neurotransmitter vesicular uptake, AADC, aromatic l-amino acid decarboxylase, CLIP, crosslink immunoprecipitation, DAT, dopamine transporter, DTSSP, 3,3′-dithiobis(sulfosuccinimidylpropionate), FDR, false discovery rate, GO, Gene Ontology, iBAQ, intensity-based absolute quantification, LFQ, label-free quantitation, MPP+, 1-methyl-4-phenylpyridinium, MPTP, 1-methyl-4-phenyl-1,2,3,6-tetrahydropyridine, PSM, peptide-spectrum match, SNpc, substantia nigra pars compacta, TH, tyrosine hydroxylase, TKO, triple α/β/γ-synuclein null mutant mice, VMAT-2, vesicular monoamine transporter 2

## Abstract

Synucleins, a family of three proteins highly expressed in neurons, are predominantly known for the direct involvement of α-synuclein in the etiology and pathogenesis of Parkinson's and certain other neurodegenerative diseases, but their precise physiological functions are still not fully understood. Previous studies have demonstrated the importance of α-synuclein as a modulator of various mechanisms implicated in chemical neurotransmission, but information concerning the involvement of other synuclein family members, β-synuclein and γ-synuclein, in molecular processes within presynaptic terminals is limited. Here, we demonstrated that the vesicular monoamine transporter 2–dependent dopamine uptake by synaptic vesicles isolated from the striatum of mice lacking β-synuclein is significantly reduced. Reciprocally, reintroduction, either *in vivo* or *in vitro*, of β-synuclein but not α-synuclein or γ-synuclein improves uptake by triple α/β/γ-synuclein–deficient striatal vesicles. We also showed that the resistance of dopaminergic neurons of the substantia nigra pars compacta to subchronic administration of the Parkinson's disease–inducing prodrug 1-methyl-4-phenyl-1,2,3,6-tetrahydropyridine depends on the presence of β-synuclein but only when one or both other synucleins are absent. Furthermore, proteomic analysis of synuclein-deficient synaptic vesicles *versus* those containing only β-synuclein revealed differences in their protein compositions. We suggest that the observed potentiation of dopamine uptake by β-synuclein might be caused by different protein architecture of the synaptic vesicles. It is also feasible that such structural changes improve synaptic vesicle sequestration of 1-methyl-4-phenylpyridinium, a toxic metabolite of 1-methyl-4-phenyl-1,2,3,6-tetrahydropyridine, which would explain why dopaminergic neurons expressing β-synuclein and lacking α-synuclein and/or γ-synuclein are resistant to this neurotoxin.

Three members of the synuclein family, α-synuclein, β-synuclein, and γ-synuclein, are involved in various important molecular processes in presynaptic terminals. Gain-of-toxic function is believed to be the main mechanism of the involvement of these proteins, α-synuclein in particular, in pathogenesis of Parkinson's disease and certain other neurodegenerative diseases collectively known as synucleinopathies, but the loss of function can also be a factor contributing to synaptic dysfunction associated with these diseases (1, 2, 3, 4, 5). However, null mutations inactivating one or even two synuclein-encoding genes have limited effects on mouse physiology. The most notable consequence of α-synuclein, γ-synuclein, or double α/γ-synuclein null mutations for nigrostriatal system function is a partial (6, 7, 8, 9) or even complete (6, 9, 10, 11, 12) resistance of dopaminergic neurons of the substantia nigra pars compacta (SNpc) to various regimens of the Parkinsonian mimetic toxin 1-methyl-4-phenyl-1,2,3,6-tetrahydropyridine (MPTP). These observations have led to the hypothesis that these two synucleins are directly involved in, and required for, the MPTP-induced degeneration of dopaminergic neurons in the SNpc.

This straightforward assumption, nevertheless, does not take into consideration a possibility that the third member of the family, β-synuclein, may play a role in the resistance of SNpc neurons to MPTP toxicity. β-synuclein is highly expressed in the same neurons as α-synuclein, including dopaminergic neurons of the SNpc, and colocalizes with α-synuclein in their presynaptic terminals (13, 14, 15). Together with the high similarity between amino acid sequences of synucleins, this suggests that when a family member is absent, it can be replaced by another one. This not only can partially compensate for the lost function but also can bring certain “added values,” that is, functional changes that manifest only when normal synaptic physiology is challenged. Indeed, increased expression of β-synuclein was observed in the midbrain of both α-synuclein and α/γ-synuclein null mutant mice, and restoration of α-synuclein expression reversed this effect (9, 12). In several experimental *in vivo* systems, expression of β-synuclein ameliorated neuronal pathology caused by overexpression of α-synuclein (16, 17, 18, 19, 20, 21). General neuroprotective abilities of β-synuclein have been attributed to inhibition of proapoptotic and activation of prosurvival signaling pathways but through unknown mechanisms (9, 16).

Here, we demonstrated that β-synuclein plays a pivotal role in the developing resistance to MPTP toxicity by SNpc dopaminergic neurons lacking the normal balance of this protein and other members of the synuclein family. This might be due, at least in part, to improved sequestering of 1-methyl-4-phenylpyridinium (MPP+) into synaptic vesicles, which is concordant with an observed ability of β-synuclein to potentiate dopamine uptake by striatal synaptic vesicles.

## Results and discussion

### SNpc dopaminergic neurons of mice lacking all three synucleins are as sensitive to subchronic MPTP toxicity as neurons of WT mice

**Figure 1:**
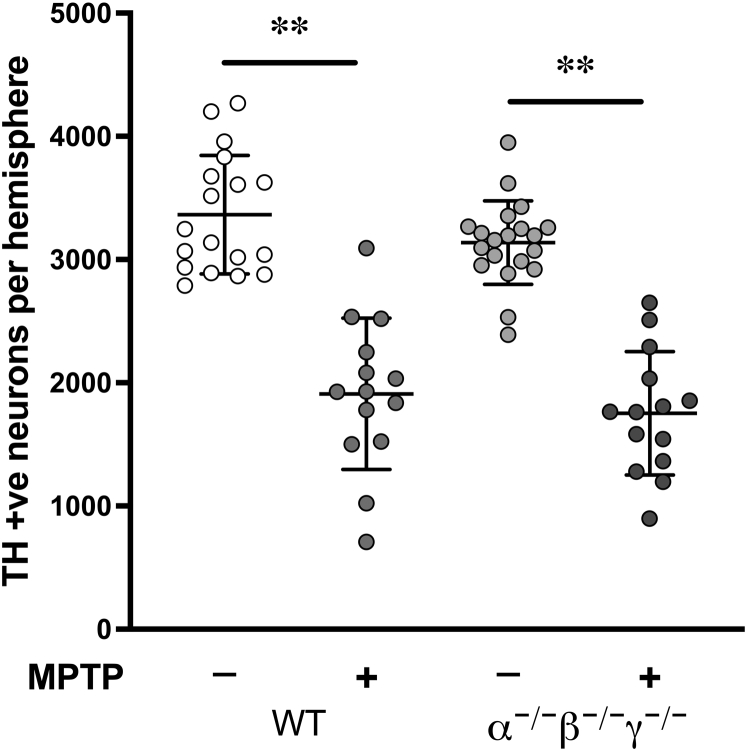
**Sensitivity of SNpc dopaminergic neurons of WT and triple synuclein deficient mice to subchronic MPTP treatment.** Scatter plot shows shows means ± SD of total number of TH-positive neurons in the SNpc of WT and triple synuclein null mutant (α^−/−^β^−/−^γ^−/−^) 5-month-old male mice. Significant loss of neurons was observed for both genotypes following MPTP treatment (∗∗*p* < 0.0001; one-way ANOVA with Sidak's multiple comparison test; n = 14–20 per genotype/treatment). No difference in the degree of neuronal loss was found between genotypes (43.2 ± 4.87% for WT mice and 44.1 ± 4.26% for TKO mice). MPTP, 1-methyl-4-phenyl-1,2,3,6-tetrahydropyridine; SNpc, substantia nigra pars compacta; TH, tyrosine hydroxylase.

A widely accepted hypothesis suggests that intrinsic toxicity of α-synuclein and γ-synuclein is a prerequisite for the demise of dopaminergic neurons in the SNpc of MPTP-treated mice. A logical extrapolation of this presumption would be that SNpc neurons of triple α/β/γ-synuclein null mutant (TKO) mice should be at least as resistant to the toxic effect of MPTP as neurons lacking α-synuclein and γ-synuclein. However, morphometric analysis of the number of tyrosine hydroxylase (TH)–positive neurons in the SNpc of WT and TKO mice treated with a subchronic MPTP regimen (10, 12) revealed the same degree of neuronal loss for both genotypes (Fig. 1). This result clearly demonstrated that none of the three synucleins are required for MPTP-induced death of dopaminergic neurons in the SNpc and suggests that a protecting role of β-synuclein may have in the single and double α/γ-synuclein knockout scenarios.

### Reduced *in vitro* dopamine uptake by striatal synaptic vesicles in the absence of β-synuclein

**Figure 2:**
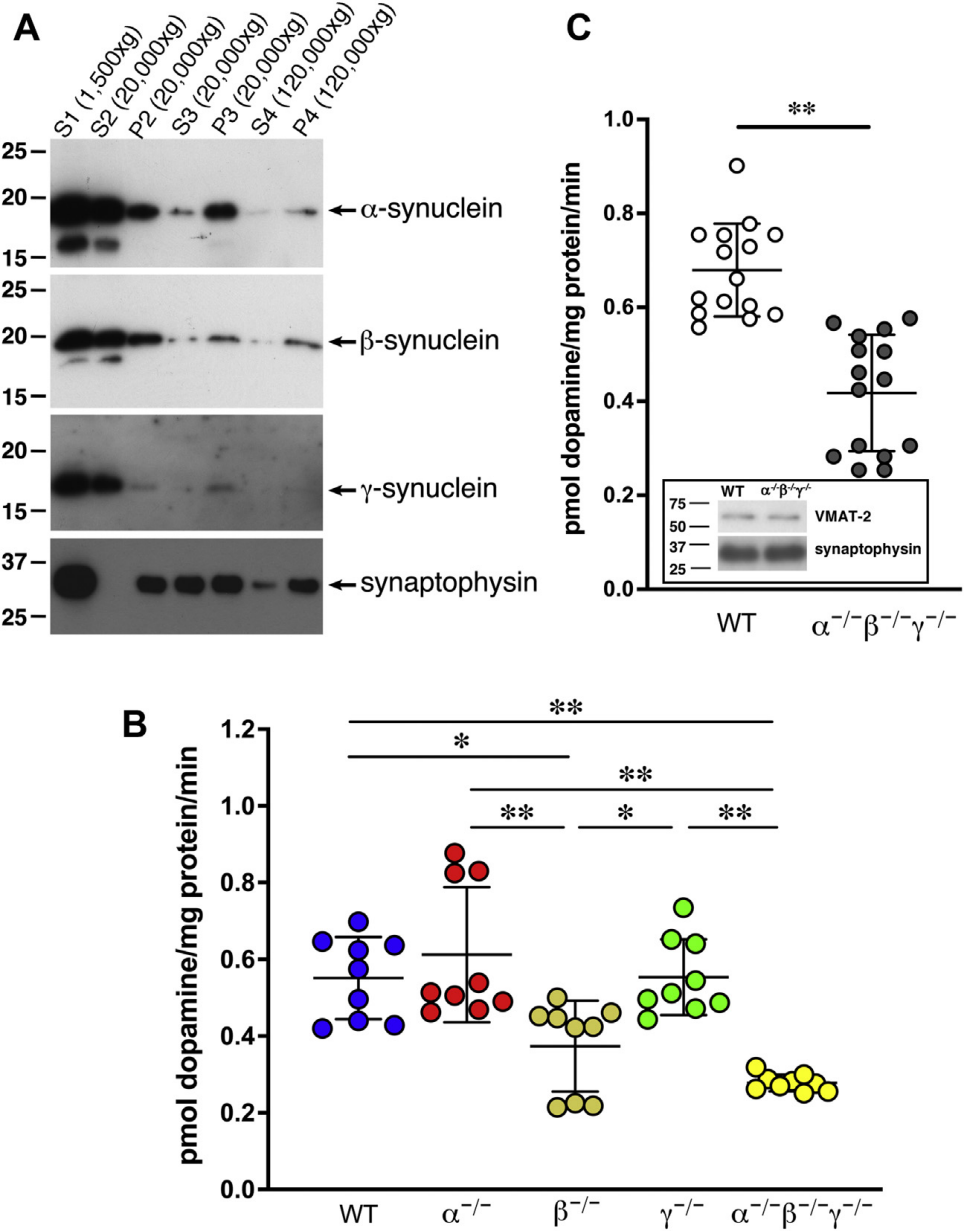
**Synucleins are associated with striatal synaptic vesicles, and vesicular dopamine uptake is reduced in the absence of β-synuclein.***A*, Western blot analysis of proteins in fractions of WT mouse striatum. Postnuclear supernatant (S1) was centrifuged at 20,000*g*, synaptosomes in the resulting pellet (P2) were lyzed by homogenization in hypotonic buffer, and again centrifuged at 20,000*g* to separate heavy synaptic membrane fraction (pellet P3) from cytosolic fraction containing small synaptic vesicle (supernatant S3). High-speed centrifugation at 120,000*g* produced the supernatant (S4) and synaptic vesicle pellet (P4). Positions and sizes (in kilodalton) of nearest protein markers are shown on the *left*. *B*, scatter plot shows means ± SD of dopamine uptake by synaptic vesicles isolated from the striatum of 13-month-old female WT, α-synuclein (α^−/−^), β-synuclein (β^−/−^), γ-synuclein (γ^−/−^) null mutant and TKO mice (∗*p* < 0.05, ∗∗*p* < 0.01; one-way ANOVA with Sidak's multiple comparison test; n = 9 for each genotype from three independent experiments). *C*, scatter plot shows means ± SD of dopamine uptake by synaptic vesicles isolated from the striatum of 5-month-old male WT and TKO (α^−/−^β^−/−^γ^−/−^) mice (∗∗*p* < 0.0001; unpaired *t* test, n = 15 for both genotypes from three independent experiments). A representative Western blot in the inset illustrates equal amounts of VMAT-2 in vesicular fractions isolated from striata of WT and TKO mice. Positions and sizes (in kilodalton) of nearest protein markers are shown on the *left*. TKO, triple α/β/γ-synuclein null mutant mice; VMAT-2, vesicular monoamine transporter 2.

The sensitivity of dopaminergic neurons to MPTP depends on the ability of its active toxic metabolite, MPP^+^, to enter the cell *via* the dopamine transporter (DAT) and to avoid being sequestered in synaptic vesicles by a vesicular monoamine transporter 2 (VMAT-2)–driven mechanism (22, 23, 24). Thus, dopaminergic neurons with a higher DAT/VMAT-2 ratio, such as SNpc neurons, are more sensitive to MPTP than neurons with a lower ratio, for instance, ventral tegmental area neurons. We have previously demonstrated that the function of DAT in synapses of SNpc neurons is not affected by the absence of either α-synuclein and γ-synuclein or all three synucleins (25, 26). This was further confirmed by studies of *in vitro* dopamine uptake by synaptosomes isolated from the striatum of WT and TKO mice that revealed no difference in this DAT-dependent uptake between the two animal groups (Fig. S1). These observations suggest that increased sensitivity to MPTP toxicity of midbrain dopaminergic neurons of TKO mice comparing with neurons deficient in α-synuclein, γ-synuclein, or both these synucleins may be related to attenuated VMAT-2–dependent MPP^+^ uptake by vesicles lacking these proteins. Consistent with the well-documented ability of synucleins to interact with various biological and synthetic membranes, particularly with high curvature vesicles (27, 28, 29), we showed that all three family members are present in the synaptic vesicle fraction despite being predominantly cytosolic proteins in the striatum of WT mice (Fig. 2*A*). It should be noted that lower than for other two synucleins, abundance of γ-synuclein in the P2 and consequently further fractions is due to more restricted expression of this family member in the mouse brain, that is, it is expressed in the substantia nigra but not in other brain regions with synaptic contacts in the striatum (30), and therefore, many striatal vesicles, mainly those originated from nondopaminergic terminals, lack γ-synuclein but contain α-synuclein and β-synuclein because of their expression in virtually all brain neurons. To check whether vesicle-associated synucleins affect VMAT-2–dependent uptake, we compared the ability of synaptic vesicles isolated from the striatum of TKO and single synuclein null mutant mice to take up ^3^H-dopamine. It has previously been shown that the basal level of cytosolic dopamine in dopaminergic neurons is below 0.1 μM, which is the detection limit of the intracellular patch electrochemistry technique (31). Therefore, to imitate physiological conditions, uptake was measured with 10 nM dopamine in the reaction mixture. We observed a significant 32.2 ± 7.15% reduction in tetrabenazine-sensitive (*i.e.*, VMAT-2-dependent) dopamine uptake by synaptic vesicles isolated from the striatum of β-synuclein null mutant female mice, whereas for vesicles isolated from the striatum of α-synuclein or γ-synuclein null mutant mice, this uptake was not different from the uptake by vesicles isolated from the striatum of WT mice (Fig. 2*B*). Higher, although not statistically significantly higher, degree of reduction, 49.6 ± 1.32%, was found for vesicles lacking all three synucleins. The latter observation was confirmed in experiments where uptakes by synaptic vesicles isolated from the striatum of 5-month-old male mice (38.5 ± 4.71% reduction; Fig. 2*C*) or 14-month-old male (39.2 ± 1.29% reduction) were compared, suggesting that the effect is not age dependent or sex dependent. Further analysis demonstrated decreased *V*_max_ (9.97 *versus* 12.29) and *K*_*M*_ (117.4 *versus* 196.1) values for this uptake in the absence of synucleins. These changes were not because of a decrease in either the number of synaptic vesicles in dopaminergic synapses or levels of VMAT-2 in the striatum of TKO mice (25) and in synaptic vesicles isolated from the striatum of TKO mice (Fig. 2*C*; inset).

Reduced dopamine uptake efficiency of synuclein-depleted synaptic vesicles and the resultant decrease of the vesicular dopamine pool explain the attenuated response of TKO mice to amphetamine observed in our previous study (25). In theory, this functional deficiency should also cause accumulation of free cytosolic dopamine within the presynaptic terminals because TH activity and dopamine reuptake by DAT are unaffected in the striatum of TKO mice (25). To prevent toxicity of free cytosolic dopamine, these mice utilize a compensatory increase of its degradation as suggested by increased striatal 3,4-dihydroxyphenylacetic acid/dopamine ratio (25).

### β-synuclein rescues SNpc dopaminergic neurons from toxic effect of MPTP but only in mice lacking other synucleins

**Figure 3:**
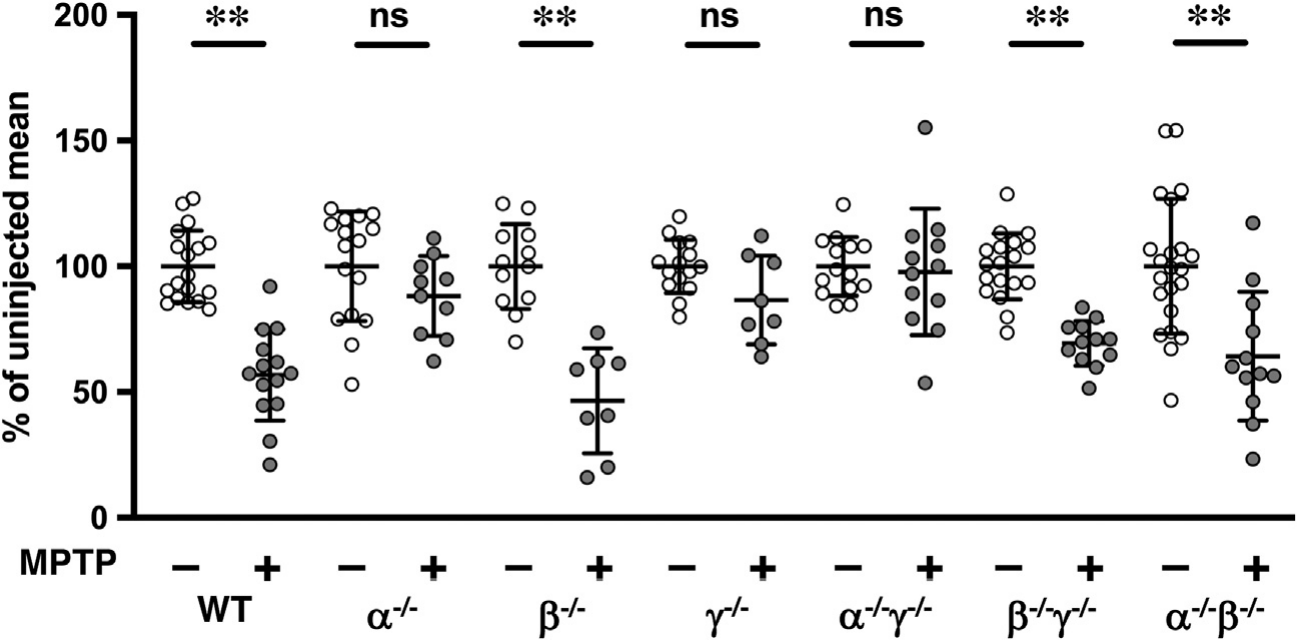
**β-synuclein is pivotal for acquiring MPTP resistance by dopaminergic neurons lacking other synucleins.** Scatter plot shows means ± SD of the number of TH-positive neurons in the SNpc of WT, single (α^−/−^, β^−/−^, γ^−/−^) and double (α^−/−^γ^−/−^, β^−/−^γ^−/−^, α^−/−^β^−/−^) synuclein null mutant mice following subchronic administration of MPTP (n = 8–14 per genotype) expressed as percent of the number of neurons in control vehicle–injected animals of the same genotype (∗∗*p* < 0.0001, one-way ANOVA with pairwise uncorrected Fisher's least significant difference test). MPTP, 1-methyl-4-phenyl-1,2,3,6-tetrahydropyridine; SNpc, substantia nigra pars compacta; TH, tyrosine hydroxylase.

It is feasible that synucleins differ in their ability to potentiate vesicular uptake of dopamine and other structurally similar molecules, for example, MPP^+^. Thus, functional substitution for the loss of a family member(s) by another, more efficacious member might significantly affect both vesicular dopamine uptake and sensitivity of dopaminergic neurons to MPTP toxicity. The resistance of α/γ-synuclein–deficient ((12) and Fig. 3) neurons, but sensitivity of α/β/γ-synuclein–deficient (Fig. 1) neurons to this drug, suggest that within the family only β-synuclein can efficiently potentiate vesicular uptake of MPP^+^. This is consistent with observation that the absence of β-synuclein but not α-synuclein or γ-synuclein reduces dopamine uptake by striatal synaptic vesicles isolated from null mutant mice (Fig. 2*B*). To confirm the pivotal role of β-synuclein in potentiating the efficiency of vesicular uptake, we first compared the sensitivity of SNpc dopaminergic neurons to subchronic MPTP administration in three groups of β-synuclein–deficient mice and in three groups of mice lacking the other two synucleins singularly or in combination. In single β-synuclein and both double β/γ-synuclein and α/β-synuclein null mutant mice, these neurons were sensitive to the drug to approximately the same degree as neurons of WT mice (Fig. 3). In contrast, this protocol of MPTP administration did not cause loss of dopaminergic neurons in the SNpc of α-synuclein, γ-synuclein, and α/γ-synuclein null mutant mice (Fig. 3), consistent with previous observations (10, 12).

### Proteomes of striatal synaptic vesicles containing all three synucleins or just β-synuclein are markedly different from the proteome of vesicles lacking all three synucleins

A possible explanation of results obtained in our *in vitro* and *in vivo* experiments described previously is a different protein composition of striatal synaptic vesicles lacking β-synuclein and those containing this protein, particularly in the absence of other synucleins that do not promote the uptake but compete with β-synuclein for binding to the membrane of synaptic vesicles. Indeed, β-synuclein is substantially more abundant on the striatal synaptic vesicles lacking other two synucleins, that is, on vesicles isolated from α/γ-synuclein null mutant mice than on vesicles isolated from WT mice (Fig. S2). This competition explains why WT mice are not resistant to MPTP toxicity despite the presence of β-synuclein. To test the aforementioned suggestion, proteomes of striatal synaptic vesicles from synuclein-free TKO mice were compared with proteomes of striatal synaptic vesicles from mice expressing only β member of the synuclein family, that is, α/γ-synuclein null mutant mice, and with proteomes of striatal synaptic vesicles from WT mice. Synaptic vesicles (fraction P4) were prepared from striata of four mice per genotype in each of three independent isolation experiments, and the protein composition of resulting nine samples was analyzed by LC–MS/MS followed by bioinformatics assessment of obtained data as described in the Experimental procedures section. Extended Proteins Identification Reports containing proteins and peptides identification details and Extended peptide-spectrum match (PSM) Reports for details about PSMs can be downloaded from the Mendeley Data site (https://data.mendeley.com/datasets/26n7cd5dzr/2; https://doi.org/10.17632/26n7cd5dzr.2).

The final curated list of proteins differentially represented in striatal synaptic vesicles of TKO *versus* α/γ-synuclein null mutant and WT mice (for inclusion criteria, see the Experimental procedures section) contained 83 proteins (Table 1 and Fig. S3). As expected, majority (*i.e.*, 50) of these proteins have been identified as constituents of the synaptic vesicle proteome in at least one of the previous MS studies (32, 33, 34, 35, 36, 37, 38). Moreover, all proteins in the curated list, independently of what functional group they belong to, showed the same direction of changes when their levels in TKO samples were compared with levels in β-synuclein–positive samples, that is, either α/γ-synuclein null mutant samples or WT samples, although for 34 proteins, the latter comparison revealed only a trend (indicated by up or down arrows with a star (∗) in Table 1) rather than statistically significant difference. But unexpectedly, there were no proteins with statistically significant difference in representation between striatal synaptic vesicles of α/γ-synuclein null mutant and WT mice with an obvious exception for synucleins. These data confirm the previous conclusion that the absence of all three synucleins in knockout mice causes certain changes in the protein composition of synaptic vesicles (29, 37, 39) and also suggest that in the intact nervous system the presence of β-synuclein alone is sufficient for an apparent reversal of the majority if not all these changes.

**Table 1:** Proteins revealed by MS analysis as differentially represented in striatal synaptic vesicles containing β-synuclein (WT, α^+/+^β^+/+^γ^+/+^, and α/γ synuclein null mutant, α^−/−^β^+/+^γ^−/−^) and lacking all synucleins (TKO, α^−/−^β^−/−^γ^−/−^)

Gene symbol	Protein symbol	Common protein name	Burre *et al.* (32)	Morciano *et al.* (34)	Takamori *et al.* (36)	Direction of changes α^−/−^β^+/+^γ^−/−^*versus* α^−/−^β^−/−^γ^−/−^	Fold changes α^−/−^β^+/+^γ^−/−^*versus* α^−/−^β^−/−^γ^−/−^	Direction of changes α^+/+^β^+/+^γ^+/+^*versus* α^−/−^β^−/−^γ^−/−^	Fold changes α^+/+^β^+/+^γ^+/+^*versus* α^−/−^β^−/−^γ^−/−^
Synaptic vesicle resident proteins									
Rtn1	RTN1	Reticulon-1	√		√	⇧	1.87	⇧ ∗	1.73
Scamp5	SCAM5	Secretory carrier-associated membrane protein 5	√		√	⇧	1.80	⇧	1.79
Thy1	THY1	Thy1	√		√	⇧	1.74	⇧	2.16
Syp	SYPH	Synaptophysin	√	√	√	⇧	1.51	⇧	1.46
Rab3a	RAB3A	Rab3a	√	√	√	⇧	1.42	⇧	1.60
Atp6v0a1	VPP1	Membrane subunit A1 of vesicular V-type proton ATPase	√	√	√	⇧	1.41	⇧	1.40
Dnajc5	DNJC5	CSPalpha	√		√	⇧	1.41	⇧	1.84
Sv2a	SV2A	SV2A	√	√	√	⇧	1.35	⇧	1.35
Atp6v0d1	VA0D1	Membrane subunit D1 of vesicular V-type proton ATPase	√	√	√	⇧	1.34	⇧	1.42
Sv2b	SV2B	SV2B	√	√	√	⇧	1.32	⇧	1.33
Syt1	SYT1	Synaptotagmin 1	√	√	√	⇧	1.29	⇧	1.30
Vamp2	VAMP2	VAMP2—vSNARE			√	⇧	1.28	⇧	1.31
Syngr1	SNG1	Synaptogyrin 1	√		√	⇧	1.26	⇧	1.30
Atp6v1h	VATH	Cytosolic subunit H of vesicular V-type proton ATPase	√		√	⇧	1.25	⇧	1.35
Syn2	SYN2	Synapsin 2	√	√	√	⇩	(−) 1.48	⇩	(−) 1.39
Syn1	SYN1	Synapsin 1	√	√	√	⇩	(−) 1.61	⇩	(−) 1.45
SNARE complex (tSNARE and interacting proteins)									
Stx1b	STX1B	Syntaxin 1B—tSNARE	√	√	√	⇧	1.46	⇧ ∗	1.26
Snap25	SNP25	SNAP25—tSNARE	√		√	⇧	1.31	⇧ ∗	1.31
Stx1a	STX1A	Syntaxin 1A—tSNARE	√		√	⇧ ∗	1.17	⇧	1.36
Cplx1	CPLX1	Complexin-1—SNARE complex interacting protein			√	⇩	(−) 1.48	⇩ ∗	(−) 1.31
Clathrin-mediated endocytosis (CME) and clathrin-independent endocytosis (CIE)									
Cltb	CLCB	Clathrin light chain B				⇧	2.06	⇧	2.03
Clta	CLCA	Clathrin light chain A				⇧	1.83	⇧	1.69
Cltc	CLH1	Clathrin heavy chain 1	√	√	√	⇧	1.56	⇧	1.67
Ap2b1	AP2B1	AP2B1	√	√	√	⇧	1.32	⇧ ∗	1.29
Ap2a2	AP2A2	AP2A2	√	√	√	⇧	1.24	⇧ ∗	1.10
Ap2a1	AP2A1	AP2A1	√	√	√	⇧	1.24	⇧ ∗	1.18
Ap2s1	AP2S1	AP2S1–AP-2 complex subunit sigma			√	⇧ ∗	1.17	⇧	1.51
Ap1b1	AP1B1	AP1B1—AP-1 complex subunit beta-1			√	⇩	(−) 1.24	⇩ ∗	(−) 1.19
Dnm1	DYN1	Dynamin-1	√	√	√	⇩	(−) 1.33	⇩ ∗	(−) 1.30
Amph	AMPH	Amphiphysin			√	⇩	(−) 1.41	⇩	(−) 1.59
Pacsin1	PACN1	Syndapin-1 (protein kinase C and casein kinase substrate in neurons protein 1)				⇩	(−) 1.62	⇩ ∗	(−) 1.43
Sh3gl2	SH3G2	Endophilin A1			√	⇩	(−) 1.88	⇩	(−) 1.86
Plasma membrane proteins									
Atp2a2	AT2A2	Sarcoplasmic/endoplasmic reticulum calcium ATPase 2			√	⇧	1.89	⇧ ∗	1.57
Atp2b2	AT2B4	Plasma membrane calcium-transporting ATPase 2			√	⇧	1.80	⇧	1.67
Slc1a2	EAA2	Excitatory amino acid transporter 2				⇧	1.79	⇧	1.72
Atp1a1	AT1A1	Sodium/potassium-transporting ATPase subunit alpha-1		√	√	⇧	1.78	⇧	1.60
Atp1b1	AT1B1	Sodium/potassium-transporting ATPase subunit beta-1	√	√	√	⇧	1.72	⇧	1.78
Gpm6a	GPM6A	Neuronal membrane glycoprotein M6-a				⇧	1.63	⇧	1.67
Atp1a3	AT1A3	Sodium/potassium-transporting ATPase subunit alpha-3	√	√	√	⇧	1.62	⇧	1.76
Ncam1	NCAM1	Neural cell adhesion molecule 1			√	⇧	1.49	⇧ ∗	1.45
Nptn	NPTN	Neuroplastin		√		⇧	1.44	⇧ ∗	1.46
Cytoskeleton and associated proteins									
Myh10	MYH10	Myosin-10				⇧	1.60	⇧	1.68
Map1a	MAP1A	Microtubule-associated protein 1A				⇧	1.25	⇧ ∗	1.12
Myl6	MYL6	Myosin light polypeptide 6				⇧	1.24	⇧ ∗	1.11
Vsnl1	VISL1	Visinin-like protein 1 (VILIP1)	√	√	√	⇩	(−) 1.35	⇩	(−) 1.23
Tppp	TPPP	Tubulin polymerization-promoting protein			√	⇩	(−) 1.45	⇩	(−) 1.33
Cfl1	COF1	Cofilin-1				⇩	(−) 1.50	⇩	(−) 1.43
Sept5	SEPT5	Septin-5				⇩	(−) 1.70	⇩ ∗	(−) 1.44
Vim	VIM	Vimentin				⇩	(−) 1.70	⇩	(−) 1.55
Nes	NEST	Nestin				⇩	(−) 2.29	⇩ ∗	(−) 1.55
Cytosolic and vesicle-associated metabolic enzymes									
Fasn	FAS	Fatty acid synthase			√	⇧	1.55	⇧ ∗	1.44
Glul	GLNA	Glutamine synthetase	√		√	⇧	1.37	⇧	1.28
Pkm	KPYM	Pyruvate kinase	√	√	√	⇧	1.24	⇧ ∗	1.03
Ppia	PPIA	Peptidyl-prolyl *cis*-*trans* isomerase A or cyclophilin A				⇩	(−) 1.20	⇩	(−) 1.24
Pgam1	PGAM1	Phosphoglycerate mutase 1				⇩	(−) 1.28	⇩	(−) 1.23
Fkbp1a	FKB1A	Peptidyl-prolyl *cis*-*trans* isomerase FKBP1A				⇩	(−) 1.28	⇩ ∗	(−) 1.01
Gapdh	G3P	Glyceraldehyde-3-phosphate dehydrogenase, GAPDH	√	√	√	⇩	(−) 1.29	⇩ ∗	(−) 1.17
Ckb	KCRB	Creatine kinase B-type			√	⇩	(−) 1.35	⇩	(−) 1.42
Ldhb	LDHB	Lactate dehydrogenase B chain	√	√	√	⇩	(−) 1.36	⇩	(−) 1.23
Ak1	KAD1	Adenylate kinase isoenzyme 1				⇩	(−) 1.37	⇩ ∗	(−) 1.05
Tpi1	TPIS	Triosephosphate isomerase				⇩	(−) 1.56	⇩	(−) 1.49
Pgk1	PGK1	Phosphoglycerate kinase 1				⇩	(−) 1.71	⇩ ∗	(−) 1.46
Cp	CERU	Ceruloplasmin or ferroxidase				⇩	(−) 1.89	⇩	(−) 1.23
Intracellular signaling proteins									
Gnb2	GBB2	Guanine nucleotide-binding protein G(I)/G(S)/G(T) subunit beta 2	√	√	√	⇧	1.70	⇧ ∗	1.05
Gng7	GBG7	Guanine nucleotide-binding protein G(I)/G(S)/G(O) subunit gamma 7				⇧	1.60	⇧ ∗	1.48
Camk2b	KCC2B	Calcium/calmodulin-dependent protein kinase type II subunit beta			√	⇧	1.53	⇧	1.48
Gnao1	GNAO	Guanine nucleotide-binding protein G(O) subunit alpha	√	√	√	⇧	1.52	⇧	1.57
Gap43	NEUM	Neuromodulin, GAP-43				⇧	1.44	⇧ ∗	1.51
Gnb1	GBB1	Guanine nucleotide-binding protein G(I)/G(S)/G(T) subunit beta 1				⇧	1.38	⇧	1.31
Akap5	AKAP5	A-kinase anchor protein 5				⇧	1.31	⇧	1.41
Camk2a	KCC2A	Calcium/calmodulin-dependent protein kinase type II subunit alpha	√	√	√	⇧	1.27	⇧	1.26
Prkar2b	KAP3	cAMP-dependent protein kinase type II-beta regulatory subunit				⇧ ∗	1.19	⇧	1.26
Plcb1	PLCB1	Phospholipase C beta 1				⇩	(−) 1.21	⇩ ∗	(−) 1.28
Dpysl2	DPYL2	Dihydropyrimidinase-related protein 2		√		⇩	(−) 1.29	⇩	(−) 1.25
Ppp3ca	PP2BA	Protein phosphatase 3 catalytic subunit alpha				⇩	(−) 1.40	⇩ ∗	(−) 1.29
Arfgap1	ARFG1	ADP-ribosylation factor GTPase-activating protein 1				⇩	(−) 1.41	⇩ ∗	(−) 1.12
Ppp1r1b	PPR1B	Protein phosphatase 1 regulatory subunit 1B				⇩	(−) 1.48	⇩	(−) 1.45
Ppp3r1	CANB1	Protein phosphatase 3 regulatory subunit B alpha isoform 1 or calcineurin subunit B type 1				⇩	(−) 1.52	⇩	(−) 1.27
Chaperones, protein folding, and degradation									
Ywhag	1433G	14-3-3 gamma				⇩	(−) 1.20	⇩	(−) 1.20
Ywhab	1433B	14-3-3 protein beta/alpha				⇩	(−) 1.26	⇩	(−) 1.22
Hsp90ab1	HS90A	HSP 90-beta				⇩	(−) 1.39	⇩	(−) 1.26
Ywhat	1433T	14-3-3 theta			√	⇩	(−) 1.44	⇩ ∗	(−) 1.14
Rnf214	RN214	RING finger protein 214 (ubiquitin transferase)				⇩	(−) 1.83	⇩ ∗	(−) 1.22

Up and down arrows show direction of protein representation changes in a proteome of vesicles containing β-synuclein when compared with the proteome of TKO vesicles with the fold changes shown in the adjacent columns. Arrows with stars (∗) indicate that the difference is not statistically significant. See also Fig. S3.

A plausible mechanistic explanation is that the presence of β-synuclein on the surface of synaptic vesicles prevents excessive inclusion or interaction of proteins with high affinity to “synuclein-naked” vesicles, for example, endophilins, synapsins, and amphiphysin ((37, 39) and Table 1), and potentiates association with vesicles for other proteins, for example, synaptophysin, Rab3A, and subunits of the vesicular proton pump; these reciprocal changes in protein composition have functional consequences, including improved uptake of dopamine and structurally similar molecules by β-synuclein–containing striatal synaptic vesicles.

### Supplementing striatal synaptic vesicles with recombinant β-synuclein *in vitro* or *in vivo* potentiates dopamine uptake

**Figure 4:**
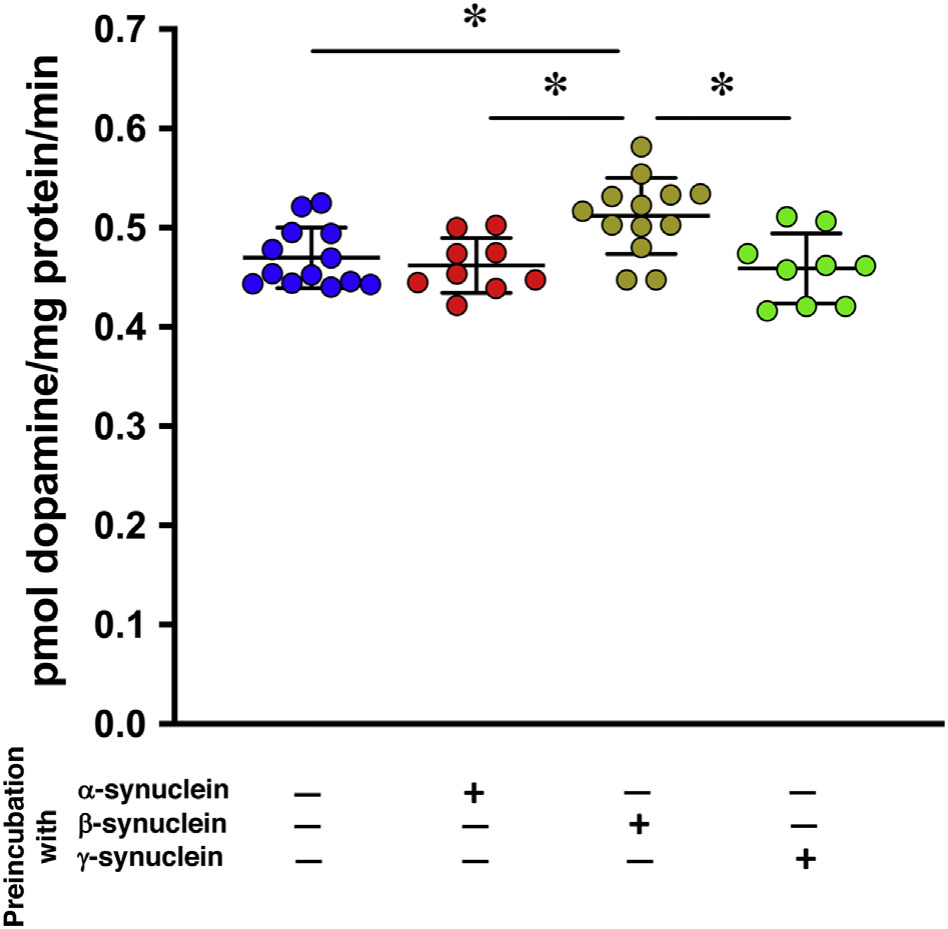
**β-synuclein potentiates vesicular dopamine uptake *in vitro*.** Effect of purified recombinant synucleins on dopamine uptake by synaptic vesicles isolated from TKO mice. Scatter plot shows means ± SD of dopamine uptake after preincubation of S3 supernatant without or with recombinant synucleins (final concentration of 20 μg/ml) followed by high-speed sedimentation of vesicles before they were used in the uptake reaction (n = 9–13 for each condition from three independent experiments, ∗*p* < 0.05, Kruskal–Wallis ANOVA with post hoc Dunn's test). TKO, triple α/β/γ-synuclein null mutant mice.

**Figure 5:**
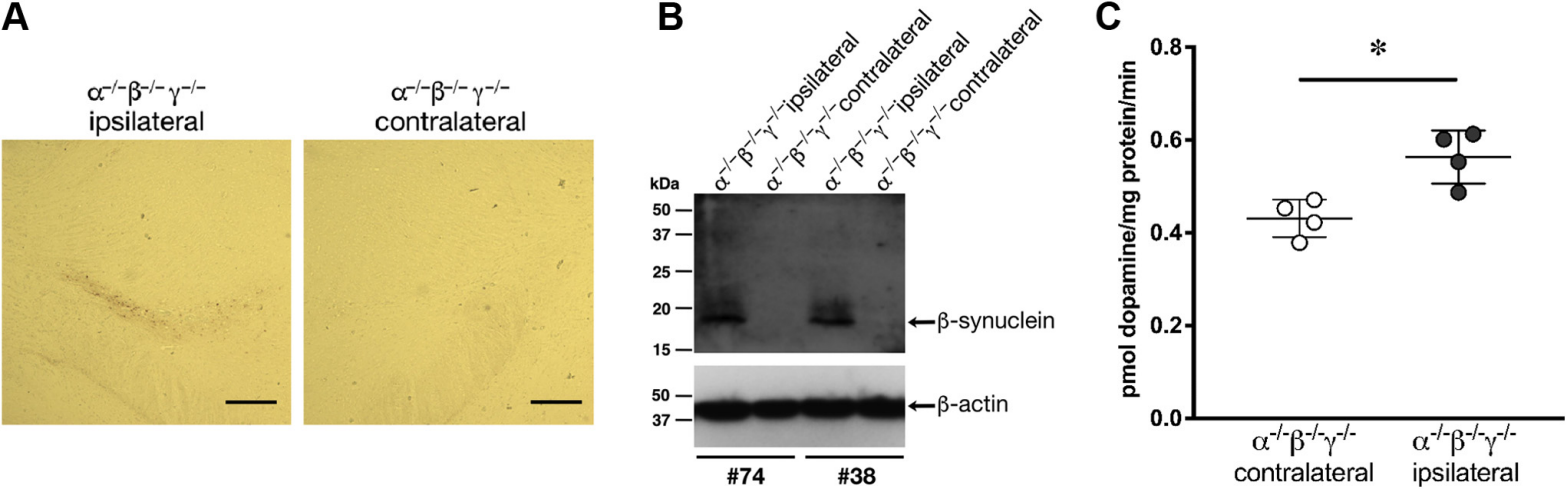
**β-synuclein potentiates vesicular dopamine uptake *in vivo*.** Analysis of dopamine uptake by synaptic vesicles from striata of TKO (α^−/−^β^−/−^γ^−/−^) mice stereotaxically injected into SNpc region with β-synuclein-expressing lentiviral vector particles. *A*, transverse paraffin sections of mouse brains were immunostained with antibody recognizing β-synuclein (recombinant rabbit monoclonal, clone EP1537Y; Abcam; 1:500). Expression of human β-synuclein was detected in the nigrostriatal tract of the ipsilateral hemisphere (injected with β-synuclein-expressing vector) but not the contralateral hemisphere (injected with control empty vector). The scale bar represents 300 μm. *B*, Western blot confirms the presence of β-synuclein in the ipsilateral but not contralateral striatum of two injected TKO mice. Positions and sizes (in kilodalton) of nearest protein markers are shown on the *left*. *C*, scatter plot shows means ± SD of dopamine uptake by synaptic vesicles isolated from the ipsilateral and contralateral striatum of six TKO animals (∗*p* < 0.0286, Mann–Whitney *U* test). SNpc, substantia nigra pars compacta; TKO, triple α/β/γ-synuclein null mutant mice.

If the aforementioned suggestion is correct, one can expect that supplementing vesicles isolated from the striatum of TKO mice with recombinant β-synuclein in the presence of soluble components of presynaptic terminals' milieu will improve dopamine uptake. Therefore, we assessed if the presence of purified recombinant synucleins would affect the dopamine uptake by striatal synaptic vesicles from TKO mice. When the vesicle-containing S3 supernatant was incubated with recombinant β-synuclein followed by spinning down synaptic vesicles (*i.e.*, obtaining P4 fraction from β-synuclein–preincubated S3 supernatant) and using them to measure dopamine uptake, a statistically significant increase was observed compared with the uptake by vesicles from a mock-incubated S3 supernatant or supernatants preincubated with recombinant α-synuclein or γ-synuclein (Fig. 4). These results are consistent with previous evidence (Fig. 2*B*) for the importance of β-synuclein but not two other family members for efficient dopamine uptake by striatal synaptic vesicles. Preincubation of isolated vesicles (*i.e.*, from P4 fraction) with the recombinant β-synuclein before adding ^3^H-dopamine did not affect the uptake (Fig. S4), suggesting that the presence of certain proteins contained within the S3 supernatant is required for efficient potentiation of synaptic vesicle dopamine uptake by β-synuclein. Next, we attempted to restore β-synuclein expression in midbrain neurons of TKO mice using *in vivo* lentiviral delivery of an expression construct. Stereotaxic injection of viral particles into the SNpc region resulted in β-synuclein expression and its transport *via* the nigrostriatal tract to the dorsal striatum where the protein could be detected by Western blotting (Fig. 5, *A* and *B*). Vesicular dopamine uptake was significantly higher in vesicles isolated from the ipsilateral than from contralateral striatum (Fig. 5*C*), suggesting that β-synuclein potentiated this process.

### Proteome analysis of striatal synaptic vesicles from TKO mice pulled down *via* added recombinant β-synuclein

In an attempt to provide an insight into how supplementing with exogenous β-synuclein improves dopamine uptake by striatal synaptic vesicles from synuclein-free mice, we assessed the proteome of β-synuclein–bound vesicles by a combination of the crosslink immunoprecipitation (CLIP) and MS techniques. Synaptic vesicle–containing S3 supernatant obtained from the striata of TKO mice was preincubated with Strep-tagged human β-synuclein followed by incubation with a cleavable crosslinking agent 3,3′-dithiobis(sulfosuccinimidylpropionate) (DTSSP) and subsequent high-speed centrifugation to spin down synaptic vesicles. Synaptic vesicles isolated from the same S3 supernatant sample preincubated without β-synuclein but similarly crosslinked were used as a nonspecific binding control (for details and the rationale for using this control, see the Experimental procedures section). Membranes of both control and β-synuclein–bound vesicles were lyzed in a nonionic detergent solution, and protein complexes containing β-synuclein were pulled down using Tactin magnetic beads that have high affinity for Strep-tagged proteins. Proteins incorporated in these complexes were eluted from the beads by breaking crosslinking bonds with DTT, leaving Strep-tagged β-synuclein attached to beads. Protein composition of the resulting CLIP proteome was analyzed by MS. Proteins identified by the presence of at least two unique peptides in the β-synuclein-CLIP and undetectable in the control CLIP were further filtered using the CRAPome tool (crapome.org) to remove common nonspecific proteins often present in MS immunoprecipitation experiments.

**Figure 6:**
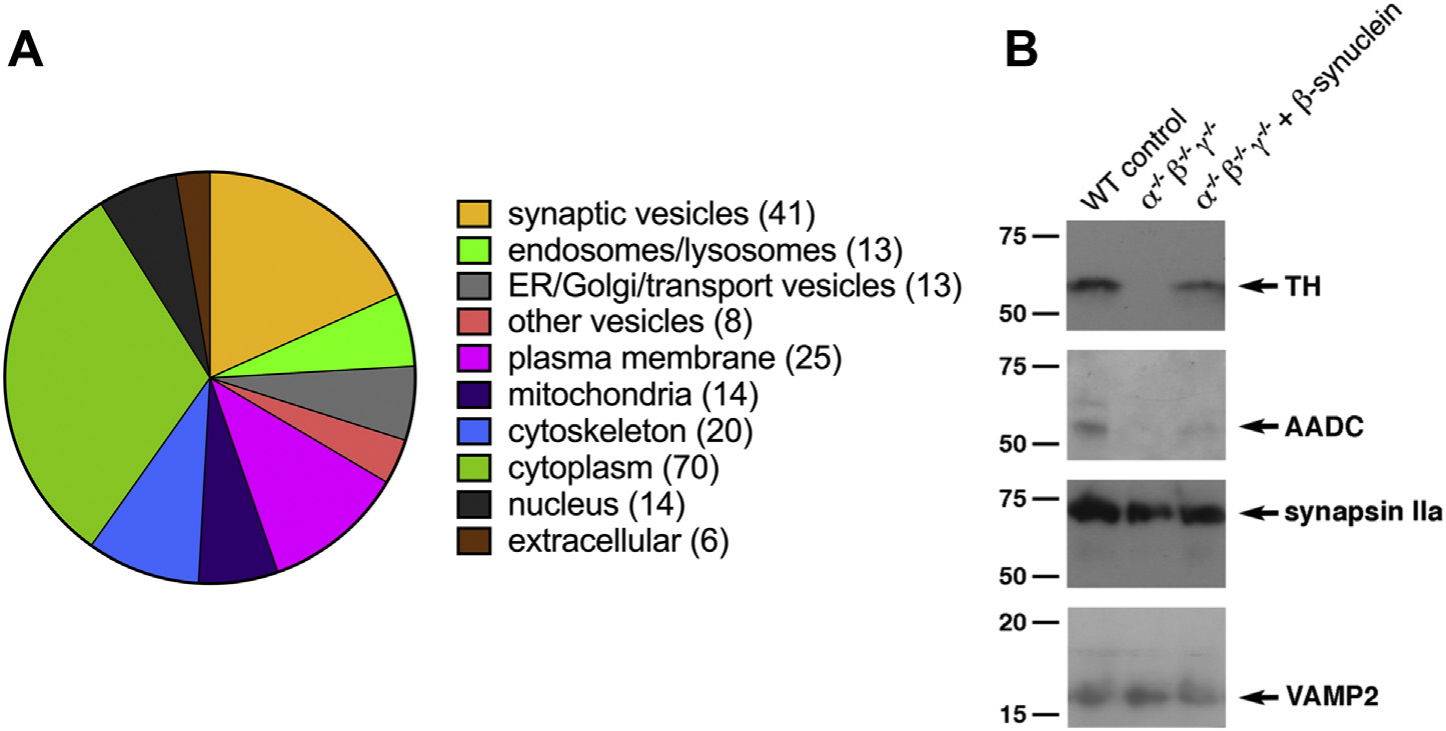
**Proteins enriched in β-synuclein-CLIP proteome.***A*, a pie chart illustrates representation in the β-synuclein-CLIP proteome of proteins with predominant localization and/or function in specific cellular compartments. The number of proteins in each group is shown in *parentheses*. See also Table S2. *B*, Western blots of protein samples from the β-synuclein-CLIP (α^−/−^β^−/−^γ^−/−^ + β-synuclein), control CLIP (α^−/−^β^−/−^γ^−/−^) and a total protein lysate of WT mouse striata (WT control) probed with antibodies against two predominantly cytoplasmic proteins (TH and AADC) and two proteins predominantly associated with synaptic vesicles (VAMP2 and synapsin IIa). Positions and sizes (in kilodalton) of nearest protein markers are shown on the *left*. AADC, aromatic l-amino acid decarboxylase; CLIP, crosslink immunoprecipitation; TH, tyrosine hydroxylase.

Not surprisingly, around half of the 224 proteins included in the final analysis (thereafter, β-synuclein-CLIP proteome; for list of protein hits sorted by the number of identified unique peptides, see Table S1) were proteins with predominant localization to, or function at, neuronal membranous structures and synaptic vesicles in particular (Fig. 6*A* and Table S2). Presynapse (Gene Ontology [GO]: 0098793) appeared to be the top cellular component with a false discovery rate (FDR) of 3.44e-27. Mechanistically, proteins involved in presynaptic functions were also vastly overrepresented in this β-synuclein-CLIP proteome: GO analysis revealed Synaptic Vesicle Cycle (GO: 0099504) and Neurotransmitter Transport (GO: 0006836) as the most represented biological processes with FDR of 4.91e-12 and 3.35e-11, respectively. Similarly, synaptic vesicle cycle (MMU4721; FDR 4.85e-12) appeared to have the highest representation between Kyoto Encyclopedia of Genes and Genomes pathways, as well as transmission across chemical synapse (MMU112315) and neurotransmitter release cycle (MMU112310)—between reactome pathways (FDR 6.88e-12 and 5.71e-12, respectively).

### Two cytosolic proteins playing key role in dopamine turnover in synaptic terminals are attracted to synaptic vesicles by β-synuclein

Various proteins, which are not intrinsic constituents of synaptic vesicles and dispensable for their function at any stage of their presynaptic cycle, have been previously detected in the brain synaptic vesicle fraction. It has been suggested that some of these proteins can modulate vesicular uptake of dopamine, for example, by increasing local availability of ATP in the case of glycolytic enzymes (34) or direct modulation of VMAT-2 activity (40, 41, 42). A number of principally cytosolic proteins has been identified in our analysis of the β-synuclein-CLIP proteome, and some of them could be considered as potential candidates for augmentation of synaptic dopamine uptake. For example, this proteome contains cytosolic enzymes TH and aromatic l-amino acid decarboxylase (AADC; also known as l-3,4-dihydroxyphenylalanine decarboxylase), and the appearance of these proteins in P4 synaptic vesicle fraction following preincubation of S3 fraction with recombinant β-synuclein has been confirmed by the Western blot analysis (Fig. 6*B*). Consistently, in our total striatal synaptic vesicle proteome analysis described previously, AADC was completely absent in all studied samples from TKO mice, whereas it has been detected, although below validation levels, in samples from WT and α/γ-synuclein null mutant mice.

TH and AADC are not only involved in presynaptic dopamine production but also can form a transient complex with VMAT-2, which creates spatial coupling of dopamine synthesis and loading into synaptic vesicles (43, 44). Although this coupling cannot explain neither the improved ^3^H-dopamine uptake in the *in vitro* assay nor the improved synaptic vesicle uptake of MPP^+^ in the striatum of MPTP-treated mice, the β-synuclein–triggered formation of the TH/AADC/VMAT-2 complex might have an allosteric effect on the transporter function.

### How β-synuclein might improve vesicular uptake and why it protects SNpc compacta dopaminergic neurons only in the absence of one or both other synucleins?

Although the role of TH/AADC/VMAT-2 complex is an attractive scenario, a contribution from other proteins interacting with the vesicle-bound β-synuclein cannot be excluded, and it is feasible that improved function of VMAT-2 in the presence of β-synuclein is the result of a cumulative effect of several multiprotein interactions rather than of any one particular complex. Indeed, our data suggest that β-synuclein, *via* interaction with a number of vesicular and cytosolic proteins, can potentiate formation of various molecular complexes on the surface of synaptic vesicles. This is similar to α-synuclein, which is known to interact with a variety of proteins and function as a chaperone or scaffold for the assembly of multiprotein complexes on the surface of synaptic vesicles, implicating α-synuclein in a number of molecular processes at several stages of the synaptic vesicle cycle (45, 46, 47, 48). However, potentiation of vesicular uptake is not included in the list of possible functions of α-synuclein or γ-synuclein, and therefore, they are unable to compensate for the loss of this particular function of β-synuclein.

All three members of the synuclein family can interact with phospholipids at the outer surface of synaptic vesicles *via* their conserved N-terminal repeat domain, but the number of functional multiprotein complexes each member can form on a given vesicle is limited. Moreover, results of the recent study (48) suggested that synucleins modulate each other's binding to synaptic vesicle–like membranes possibly *via* formation of heteromeric complexes. Such synuclein complexes attract different combination of other proteins than single synuclein-based complexes (*e.g.*, β-synuclein-triggered) resulting in formation of structurally and functionally different higher-order multiprotein complexes. It is feasible that in the presence of all three synucleins (*i.e.*, in the synaptic terminals of dopamine neurons of WT animals), this competition for space and the type of forming complexes results in a limited number of β-synuclein–triggered complexes assembled at the vesicle surface. This number is not sufficient to potentiate VMAT-2–dependent uptake to the level that can efficiently sequester MPP^+^ in synaptic vesicles and thus, prevent its toxic effect. However, in the absence of one or two other family members, β-synuclein occupies vacant sites on the vesicle surface, triggering formation of more multiprotein complexes that enable efficiently potentiate vesicular uptake of dopamine and MPP^+^ and thus, making dopaminergic neurons more robust to MPTP-induced toxicity.

In conclusion, our experimental data provide evidence that of the three members of the synuclein family, only β-synuclein can potentiate VMAT-2–dependent uptake of dopamine and structurally similar molecules by synaptic vesicles. We suggest that the increased presence of β-synuclein-triggered complexes at the synaptic vesicles, and not the absence of other synucleins *per se*, explains the decreased sensitivity to MPTP toxicity of SNpc dopaminergic neurons in mice lacking α-synuclein and/or γ-synuclein.

## Experimental procedures

### Experimental animals

Generation of α-synuclein KO (from original mouse line described (30)), β-synuclein KO (from original mouse line described (49)), γ-synuclein KO (50), and α/γ-synuclein double KO mice on C57Bl6J (Charles River) background was described previously (12, 25). Homozygous α/γ-synuclein double KO and β-synuclein KO mice were crossed to generate triple heterozygous mice. Intercrossing of these mice produced founders of TKO, double KO, single KO, and WT colonies used in this study. Therefore, founders of all these colonies were either first-generation or second-generation siblings, and all experimental animals were on the same C57Bl6J genetic background. Mouse genotyping was carried out as described previously (12, 49, 50). Unless otherwise stated, 4- to 5-month-old male mice were used in all experiments. All animal work was carried out in accordance with the ARRIVE guidelines and the United Kingdom Animals (Scientific Procedures) Act (1986).

### Subchronic MPTP treatment, immunohistochemistry, and neuronal cell counts

Four-month-old male mice were injected i.p. once a day for 5 consecutive days with 30 mg/kg of MPTP (Sigma). About 21 days after the last MPTP injection animals were euthanized by phenobarbital overdose, and brains were dissected. Fixation, processing, embedding, preparing of microtome sections, and their immunostaining with antibody against TH (mouse monoclonal, clone TH-2 from Sigma; diluted 1:1000) were performed as in our previous studies (12, 50). Stereological counting of neurons was carried out as previously described for single and double synuclein null mutant mice (51, 52) by investigators blinded with respect to the sample genotype and treatment.

### Immunoblotting

SDS gel electrophoresis and Western blotting were performed as described in our previous publications (53, 54). Primary antibodies against AADC (rabbit polyclonal; Synaptic Systems; 1:1000), TH mouse monoclonal, clone 2 (mouse monoclonal, clone TH-2; Sigma; 1:5000), synaptophysin (mouse monoclonal, clone 2; BD Transduction Laboratories; 1:25,000), synapsin IIa (mouse monoclonal, clone 1; BD Transduction Laboratories; 1:5000), VAMP2 (mouse monoclonal, clone 69.1; Synaptic Systems; 1:1000), VMAT-2 (rabbit polyclonal; Sigma; 1:1000), α-synuclein (mouse monoclonal, clone 42; BD Transduction Laboratories; 1:500), β-synuclein (mouse monoclonal, clone 8; BD Transduction Laboratories; 1:5000), and γ-synuclein (rabbit polyclonal; SK23 (50); 1:1000), secondary horseradish peroxidase–conjugated antimouse or anti-rabbit antibodies (GE Healthcare; 1:3000), and ECL+ system (GE Healthcare) were used for detection of target proteins.

### Recombinant synucleins

Expression in *Escherichia coli* and purification of recombinant synucleins were performed as described previously (55). For CLIP experiments, a fragment encoding SAWSHPQFEK sequence tag was cloned in frame with the β-synuclein ORF in the pRK172 expression vector, and the same expression and purification protocols were used to produce β-synuclein with this C-terminal Strep-tag.

### Preparation of synaptosomal and synaptic vesicle fractions

All procedures were carried out at 4 °C. Dorsal striata dissected from six mice were homogenized in 1 ml of the 0.32 M sucrose; 5 mM Hepes, pH 7.4 with protease inhibitors (Complete Mini from Roche) using a glass homogenizer (if more striata were used, all volumes were scaled up accordingly). Nuclei and cell debris were sedimented by centrifugation at 1000*g* for 10 min, and the supernatant (S1) was further centrifuged at 20,000*g* for 20 min to obtain cytosolic (S2, supernatant) and crude synaptosome (P2, pellet) fractions. For synaptosomal dopamine uptake assay, the P2 pellet was resuspended by vortexing in 0.5 ml of synaptosomal uptake assay buffer (10 mM Hepes, pH 7.4; 5.6 mM glucose; 120 mM NaCl; 5 mM KCl; 1.2 mM CaCl_2_; 1.2 mM MgCl_2_; 1 mM ascorbic acid; and 10 μM pargyline). For isolation of synaptic vesicles, the P2 pellet was resuspended by vortexing in 0.5 ml of the 0.32 M sucrose, diluted with 2 ml of deionized water, homogenized in a glass/Teflon homogenizer, and left on ice for 10 min before adding 0.3 ml of 250 mM Hepes, pH 7.4, and 0.3 ml of 1 M potassium tartrate. Synaptic membranes (P3) were separated by centrifugation at 20,000*g* for 20 min, and the supernatant (S3) was centrifuged at 120,000*g* for 40 min to obtain the supernatant (S4) and synaptic vesicle pellet (P4) that was resuspended in 1 ml of vesicular uptake assay buffer (25 mM Hepes, pH 7.4; 100 mM potassium tartrate; 0.1 mM EDTA; 0.05 mM EGTA; 1.7 mM ascorbic acid; and 2 mM ATP) using a syringe fitted with the 25-gauge needle.

### Comparative analysis of synaptic vesicle proteomes

Synaptic vesicle pellets (P4) were processed for LC/MS, and results were analyzed generally as described previously (56, 57).

#### Sample preparation for proteomic analysis

Synaptic vesicle pellets were resuspended in a denaturation solution consisting of 5 M urea, 1% sodium salt of deoxycholic acid, 10% acetonitrile, 300 mM sodium chloride, and 75 mM triethylammonium bicarbonate (pH 8.5). Proteins were reduced and alkylated by 7 mM Tris (2-carboxyethyl) phosphine and 0.2% 4-vinyl pyridine in 30% isopropanol, respectively, for 30 min followed by ten times dilution in 75 mM triethylammonium bicarbonate and two-step digestion with trypsin (200 ng/μl) for 3 h at a ratio of 1:50 (w/w) at a temperature 38 °C and for 2 h at a ratio of 1:100 (w/w) at a temperature of 42 °C. Samples were acidified by formic acid to 0.5% at final concentration and centrifuged at 12,000*g* for 10 min at 10 °C to sediment deoxycholic acid. The obtained solution of peptides was desalted using mixed cation-exchange MCX (Oasis; 30 mg, 1 cc; Waters) columns. The eluted fraction dried under a vacuum with a reduced temperature of 30 °C, and pellets were solubilized in 0.5% formic acid.

#### High-resolution MS analysis

Peptides were detected by a high-resolution Q Exactive HF-X mass spectrometer equipped with NSI ion source in a positive electrospray ionizing mode in three technical replicates per each of three biological samples per genotype. Precursor ions surveyed within a range of 420 to 1200 *m/z* at a normalized resolution of *R* = 60 K. Precursors with charge state between *z* = 2+ and *z* = 6+ were isolated by quadrupole within 2 *Th* isolation window accumulated for 12 ms of integration time, or until acquisition gain control reaches 3e6 ions (trap underfill ratio allowed at 5%). Accumulated ions were triggered for the tandem MS/MS scanning and fragmented in an high-energy collision dissociation mode at a 27% normalized energy. The resulting fragment ions were accumulated for 85 ms, or until acquisition gain control reached 5e5 ions. Ions were detected within a lowest range of the first fixed mass of 11o *m/z*, and the highest mass range was determined by the charge state of precursor ion but not exceeded 2000 *m/z*.

Peptides (2 μl equal to 1 μg; concentration of 500 ng/μl) were delivered using an Ultimate 3000 UHPLC system in a nanoflow mode. Peptides were loaded on a trap column (Acclaim Pepmap 100, 100 μm × 2 cm, C18) at 10 μl/min for 4 min in 2.5% acetonitrile, 0.1% formic acid, and 0.03% acetic acid. Then peptides were separated and eluted on an analytical column (Acclaim Pepmap, 75 μm × 15 cm, C18) in 68 min linear gradient of water (mobile phase A) and acetonitrile (mobile phase B), both supplied by 0.1% formic acid and 0.03% acetic acid in a following gradient scheme (normalized to mobile phase B): 0 to 4 min, delivering/loading on the trap column, B is 3%; 4 to 7 min, B increased to 8.5%; 7 to 10 min, B increased to 11%; 10 to 46 min, B increased to 32%, 46 to 49 min, hold the B at 32%; 49 to 51 min, B increased to 37%; 51 to 52 min, rapid increase in B to 97%; 52 to 58 min, hold the B at 95%; 58 to 59.5 min, descend the B to 3%; and 59.5 to 68 min, hold the B at 3% for column reconstitution in the initial gradient condition. The flow rate was 0.3 μl/min, except the washing phase (52–58 min), when the flow rate increased to 0.45 μl/min.

#### Data analysis

Peak lists in MGF format were generated using MS Convert software (version 3.0.21210; Proteowizard) and processed using the OMSSA search algorithm embedded in the Search GUI (version 4.0.41; CompOmics) engine. Proteins were identified using the UniProtKB sequence database in FASTA format (version; July 31, 2021) restricted to *Mus musculus* taxon with 17,042 target entries and equal number of concatenated reversed decoy entries (the total number of entries was 34,084). Data visualization and curation were accomplished using the Peptide Shaker software (version 2.0.33; CompOmics). Quantitative analysis was performed using the MaxQuant software (version 2.0.1.0; Max Planck Institute of Biochemistry) with the embedded Andromeda search engine and label-free quantitation (LFQ) scheme calculating intensity-based absolute quantification (iBAQ)/LFQ values. The following search parameters were applied to both engine unless other is specified: digestion enzyme was trypsin with maximum two missed cleavages allowed; pyridilethylation of cysteine was selected as fixed modification and methionine oxidation, N and Q deamidation as a common artifact variable modification; precursor ions tolerance was set within 10 ppm, and fragment ions tolerance was set to 0.005 Da (5 mDa); deisotoping tolerance of 7 ppm was applied to fragment ions in case of MaxQuant searching; for the modified peptide, PSMs were filtered with minimum score of 40; type I error (FDR) was restricted to 1% and estimated for protein identifications, peptides, and PSMs. Only proteins with at least two peptides, one of which must be unique peptide, were chosen for the following consideration.

#### Statistical analysis

Results data (protein groups) after MaxQuant processing were uploaded in the Perseus (version 1.6.50; Max Planck Institute of Biochemistry) for the following treatment. Matrix was filtered for the identified-only proteins to remove decoy and contaminating proteins. The size of proteome was reduced to only those proteins with a frequency of at least 70% of measurements, and the rest missing values were replaced values from the normal distribution. Significance was estimated using ANOVA test at *p* < 0.05 cutoff and Benjamini–Hochberg FDR of 1%. The obtained iBAQ values were transformed to logarithmic scale, and *z*-score was calculated with reporting of mean and standard deviation values. Data were treated with ANOVA multiple-sample test with *p* < 0.01 cutoff and filtered with permutation-based FDR of 1% for 250 randomizations. Only protein hits with significant (adjusted *p* < 0.005) iBAQ difference of more than 1.2 times between TKO (α^−/−^β^−/−^γ^−/−^) and α/γ-synuclein null mutant (α^−/−^β^+/+^γ^−/−^) samples and additional four protein hits that satisfied the same criteria only for the comparison of TKO (α^−/−^β^−/−^γ^−/−^) and WT (α^+/+^β^+/+^γ^+/+^) samples were included in the final curated list.

### Crosslinking with recombinant β-synuclein, immunoprecipitation, and MS

Synaptic vesicle containing synaptosomal fraction (S3) prepared as previously from 80 TKO striata was divided into two equal aliquots (two biological replicates) and incubated with or without 50 μg recombinant Strep-tagged β-synuclein for 5 min at 30 °C. DTSSP (Pierce Biotechnology) was added to both samples to the final concentration of 5 mM. After 25 min of incubation at 30 °C, crosslinking was terminated by the addition of Tris–HCl at pH 7.5 to 50 mM, and synaptic vesicles were spun down by centrifugation at 120,000*g* for 40 min at 2 °C. Pellets were washed twice with 50 mM Tris–HCl at pH 7.7 and 150 mM NaCl. Because of these high-speed sedimentation and intensive washing steps that would remove any complexes formed by proteins unable to interact with synaptic vesicles, we opted to use as a nonspecific binding control samples crosslinked in the absence of beta-synuclein rather than carry out crosslinking with some unrelated Strep-tagged protein that is unable to interact with synaptic vesicles. Synaptic vesicle membranes were lyzed in solubilization buffer (50 mM Tris–HCl at pH 7.5, 150 mM NaCl, 1% Triton X-100 with Roche Complete Mini protease inhibitors), and lysates were incubated with 200 μl of a 50% suspension of Tactin beads (IBA Lifesciences GmbH) in solubilization buffer for 25 min at 4 °C. Beads were washed four times with solubilization buffer, and cross-linked proteins were eluted from β-synuclein (that remained attached to beads *via* its Strep tag) by incubation of beads with 50 μl of 25 mM Tris–Cl at pH 7.5; 50 mM DTT for 40 min at 37 °C with occasional mixing. Total protein content in two samples was normalized using a Quick Start Bradford protein assay (Bio-Rad). Proteins were processed for LC–MS analysis by in-solution digestion as described previously (58). Samples were sequentially reduced and alkylated in the dark with 10 mM DTT (15 min) and 25 mM iodoacetamide (30 min), respectively. Trypsin (Promega; cleavage of lysine and arginine on C-terminal side) was added to each sample to give a final 1:25 protease:protein ratio and incubated at 37 °C overnight. Postdigestion, samples were acidified with TFA followed by desalting using reversed-phase spin tips (Glygen Corp) according to the manufacturer's recommendations. Briefly, spin-tips were solvated and equilibrated with successive washes of 60% acetonitrile/0.1% formic acid and 1% acetonitrile/0.1% formic acid. Samples were then loaded onto the spin-tips and washed with 1% acetonitrile/0.1% formic acid and eluted with 60% acetonitrile/0.1% formic acid. Eluents were subsequently dried to completion using a vacuum centrifuge.

Dried peptides were solubilized in 0.1% TFA, and 2.5 μg of digested peptides were analyzed in technical duplicate. A Thermo Ultimate 3000 UHPLC system was used to separate peptides with samples loaded onto a trap column (Acclaim Pepmap 100, 100 μm I.D., 2 cm length) at 8 μl/min in loading buffer (2% acetonitrile and 0.1% TFA in water). Peptides were eluted on-line to an analytical column (Acclaim Pepmap RSLC; 50 cm × 75 μm I.D.) using a 120 min gradient separation with 4 to 45% of 80% acetonitrile/0.1% formic acid. Eluted peptides were analyzed using a Thermo LTQ Velos Orbitrap operating in positive polarity with a top ten collision-induced dissociation method. Ions for dissociation were determined from an initial 15,000 resolution MS survey scan followed by collision-induced dissociation on the ten most abundant ions. Method settings include default charge state 2, 2.0 *m/z* isolation width, a normalized collision energy of 35, activation Q value of −0.25, and activation time of 10 ms.

Raw files were analyzed using MaxQuant, version 1.5.3.8. Variable modifications of methionine oxidation, protein N-terminal acetylation, and CAMthiopropanoyl (lysine and N-terminal protein) were permitted. The reduced DTSSP crosslinker gives a +145.01975 Da addition to potential amine-reactive sites. Fixed modification of carbamidomethylation and up to four missed cleavages were also permitted. LFQ, “Re-quantify,” “match between runs,” and “iBAQ” options were all selected. Raw data were searched against the UniprotKB *M. musculus* database downloaded in November 2015. This contained 79,900 protein sequences: 16,811 from the SwissProt reviewed section and 63,089 from the TrEMBL unreviewed section. A 1% FDR at the peptide and protein level was selected. Proteins were filtered for those containing at least two unique peptides and with >2 FC intensity *versus* control pulldowns.

### Synaptic vesicle dopamine uptake

Protein concentration in the synaptic vesicle fraction was measured using Quick Start Bradford protein assay (Bio-Rad). For each uptake reaction, 100 μl of this suspension was mixed in a glass tube with 125 μl of vesicular uptake assay buffer and preincubated at 30 °C for 15 min. The uptake was initiated by adding 25 μl of 0.1 μM ^3^H-dopamine (34.6 Ci/mmol; PerkinElmer) in the same buffer and after incubation at 30 °C for 5 min terminated with 3 ml of ice-cold wash buffer (vesicular uptake assay buffer without ascorbic acid and ATP). To determine nonspecific adsorption, control tubes were incubated on ice. To assess VMAT-2–independent uptake, control reactions were performed at 30 °C in the presence of 10 μM tetrabenazine. The level of tetrabenazine-resistant uptake was similar to background levels of uptake at 4 °C and is therefore not shown. Reaction mixtures were filtered through GF/F filters. After three washes with the wash buffer, filters were air dried, placed in vials with SafeScint fluid, and radioactivity was measured using a Beckman LS6000 Scintillation Counter. For uptake kinetic studies, increasing amounts of cold dopamine (0–10 μM) was added to the reactions. Obtained data were used to calculate *V*_max_ and *K*_*m*_ by nonlinear regression method (GraphPad Prism 4.0; GraphPad Software, Inc).

### Synaptosomal dopamine uptake

Protein concentration in the crude synaptosome fraction was measured using Quick Start Bradford protein assay (Bio-Rad), and concentration of samples was adjusted to 2 mg protein per milliliter. For each reaction, 25 μl of this suspension was mixed in a glass tube with 200 μl of synaptosomal uptake assay buffer and preincubated at 30 °C for 15 min. The uptake was initiated by adding 25 μl of 9.8 μM dopamine/0.2 μM ^3^H-dopamine mixture in the same buffer and after 10 min incubation at 30 °C terminated with 3 ml of ice-cold wash buffer (synaptosomal uptake assay buffer without ascorbic acid and pargyline). To determine nonspecific adsorption, control tubes were incubated on ice. To assess DAT-independent uptake, parallel reactions were performed at 30 °C in the presence of 10 μM nomifensine. The level of nomifensine-resistant uptake was similar to background levels of uptake at 4 °C and is therefore not shown. Reaction mixtures were filtered through GF/F filters. After three washes with the wash buffer, filters were air dried, placed in vials with SafeScint fluid, and radioactivity was measured using a Beckman LS6000 Scintillation Counter.

### Stereotaxic delivery of lentivirus expressing β-synuclein

A fragment of human β-synuclein complementary DNA that included the coding region and the upstream Kozak sequence was cloned into the unique BamHI site of the pCCLsin.cPPT.PGK.eGFP.WPRE lentiviral expression vector. About 9 × 10^5^ virus transducing units were stereotaxically injected at a rate of 1.0 μl/min for 90 s into the substantia nigra (anterior–posterior = −3.0, medial–lateral = −1.2, dorsal–ventral = −4.5, relative to Bregma) of 9-month-old male TKO mice. The same amount of control virus particles prepared using “empty” vector DNA was injected into the contralateral substantia nigra. Dorsal striata from the ipsilateral and controlateral hemispheres were dissected 8 weeks after injections and used for comparing vesicular dopamine uptake. The rest of the brain was fixed, paraffin sections were prepared, and those that cut through the nigrostriatal tract were immunostained as described previously (53, 54) to detect expressed β-synuclein translocating to the synaptic terminals.

### Statistical analysis

All data are presented as means ± SD. Statistical analysis was performed using GraphPad Prism 8.2.1.

## Data availability

The MS proteomics data have been deposited to the ProteomeXchange consortium *via* the PRIDE partner repository (59) with the dataset identifiers PXD027786 (Username: reviewer_pxd027786@ebi.ac.uk; Password: G4MSnSg0) for comparing proteomes of total striatal synaptic vesicle fractions obtained from WT, TKO, and α/γ-synuclein null mutant mice and PXD027667 (Username: reviewer_pxd027667@ebi.ac.uk; Password: 7d2MZZgv) for analysis of β-synuclein-CLIP proteome. Other data generated during this study are included in this published article and its supporting information files.

## Supporting information

This article contains supporting information.

## Conflict of interest

The authors declare that they have no conflicts of interest with the contents of this article.
