# Supplementary material for: β-synuclein potentiates synaptic vesicle dopamine uptake and rescues dopaminergic neurons from MPTP-induced death in the absence of other synucleins
Source: J Biol Chem. 2021 Nov 2;297(6):101375. doi: 10.1016/j.jbc.2021.101375 (PMC8633583; doi:10.1016/j.jbc.2021.101375)
Supplement: Supplemental Figures S1–S4 [file mmc1.docx]

**β-synuclein potentiates synaptic vesicle dopamine uptake**

**and rescues dopaminergic neurons from MPTP-induced**

**death in the absence of other synucleins**

Natalia Ninkina, Steven J. Millership, Owen M. Peters, Natalie Connor-Robson, Kirill Chaprov, Arthur T. Kopylov, Alex Montoya, Holger Kramer, Dominic J. Withers and Vladimir L. Buchman

**List of the material included**

Supplementary Figures S1 – S4

Supplementary Tables S1 and S2 submitted as separate Excel files (SupplTableS1.xlsx and SupplTableS2.xlsx)

S1


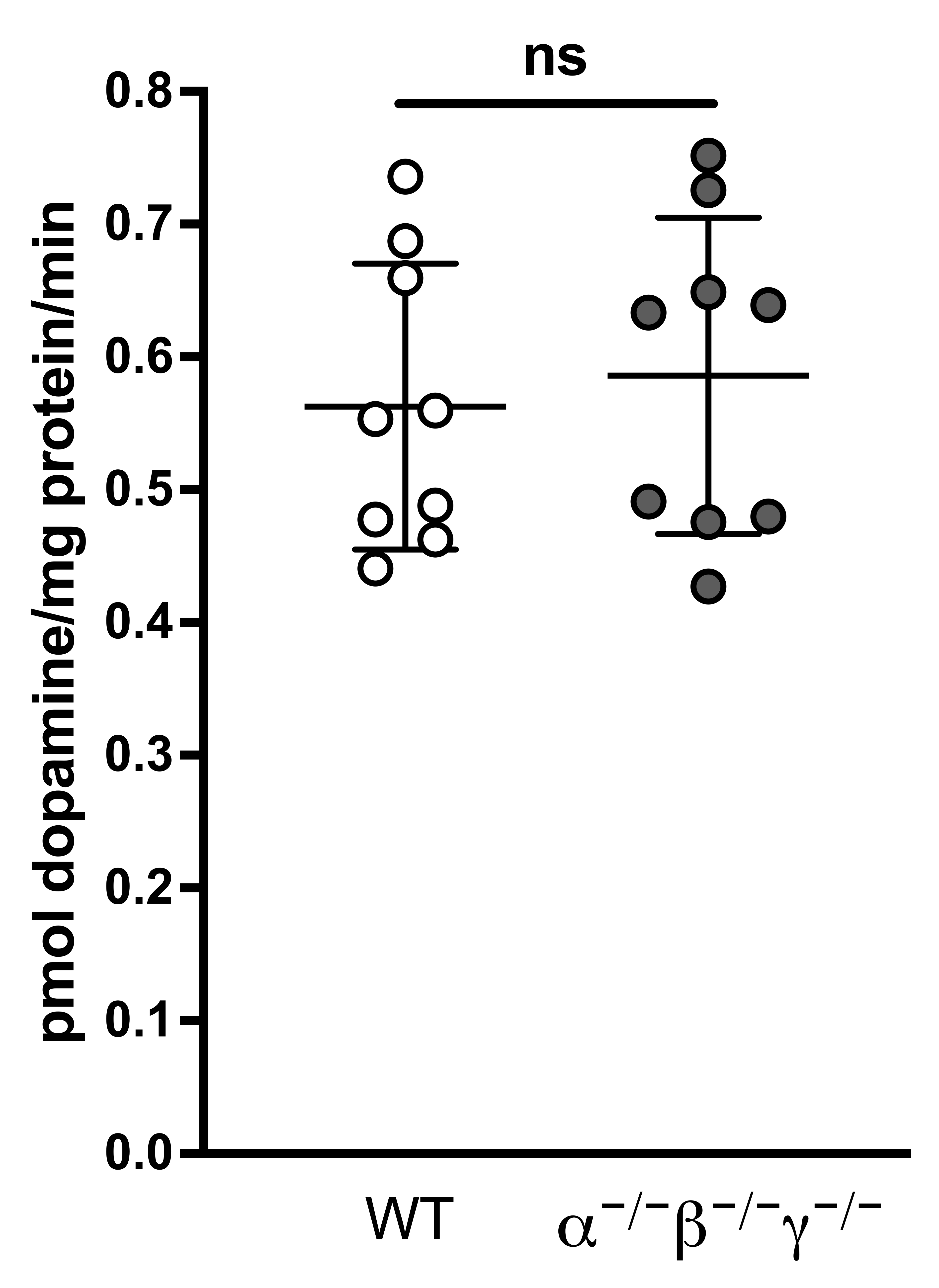


**Fig. S1.** **Dopamine uptake by striatal synaptosomes.** Scatter plot shows means±SD of dopamine uptake by synaptosomes isolated from the striatum of wild type (WT) and TKO (α^−/−^β^−/−^γ^−/−^) mice. No statistically significant differences between the groups were found (Kolmogorov-Smirnov test, n=9 for both genotypes, 3 independent experiments).


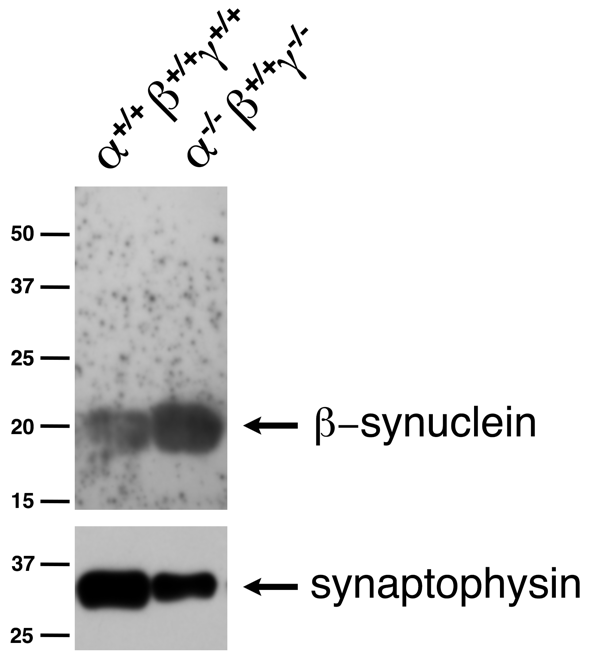


**Figure S2.** **Overrepresentation of β-synuclein on synaptic vesicles lacking other members of the synuclein family.** A Western blot of protein lysates of the purified striatal synaptic vesicles from wild type (α^+/+^β^+/+^γ^+/+^) and α/γ-synuclein null mutant (α^−/−^β^+/+^γ^−/−^) mice probed with an antibody specific to β-synuclein and re-probed with an antibody specific to synaptophysin. Positions and sizes (in kDa) of nearest protein markers are shown on the left.

S2


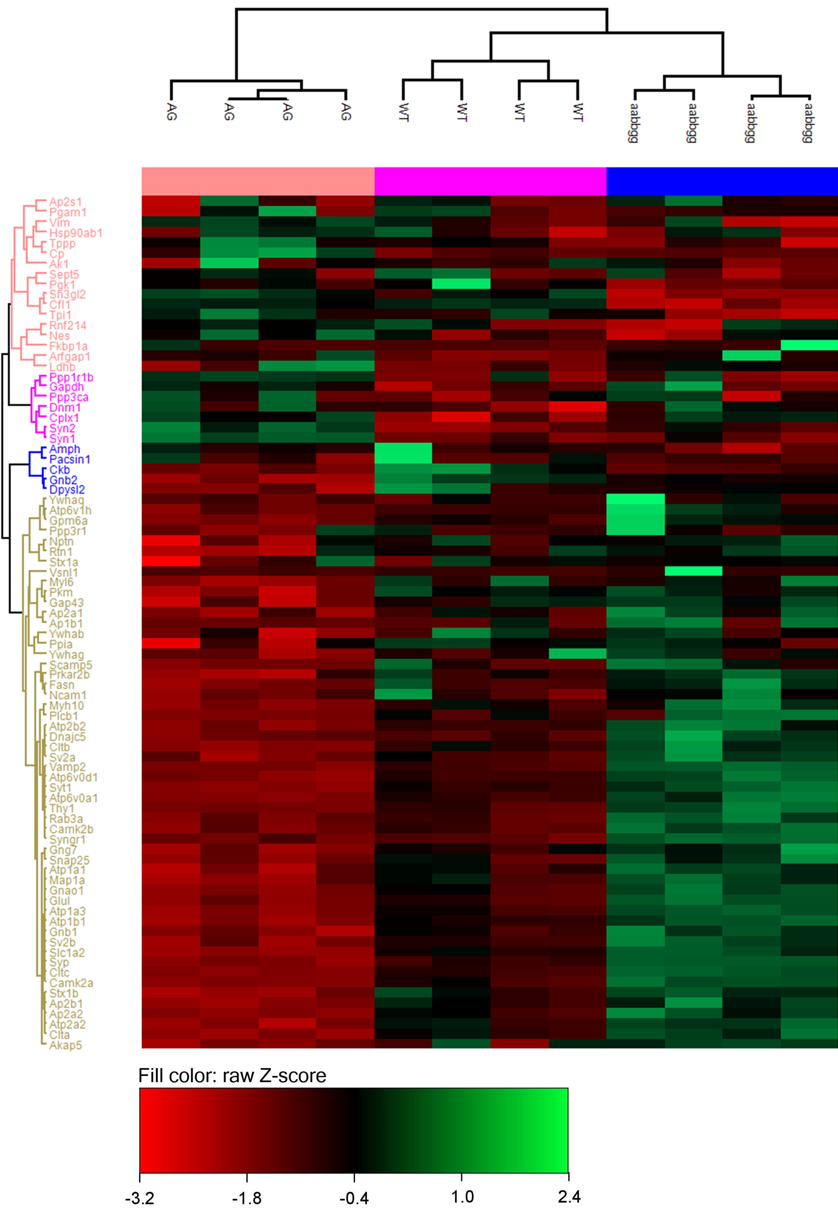


**Figure S3. Differences in striatal vesicle proteomes of TKO, α/γ-synuclein null mutant and WT mice.** Heatmap illustrates statistically significant differences (three-way comparison, p <0.01, ANOVA test) in representation of 83 proteins in the striatal vesicles of mice lacking all three synucleins (TKO, aabbgg), mice expressing only β member of the family (α/γ-synuclein null mutant, AG) and mice expressing all three synucleins (WT). Only protein hits that in our proteomic analysis showed significant (adjusted p <0.005) iBAQ difference of more than 1.2 times in pairwise comparisons between TKO and α/γ-synuclein null mutant samples and additional 4 protein hits that satisfied the same criteria only for the comparison of TKO and WT samples (i.e., the same set of proteins that is shown in Table 1) are included in the heatmap.


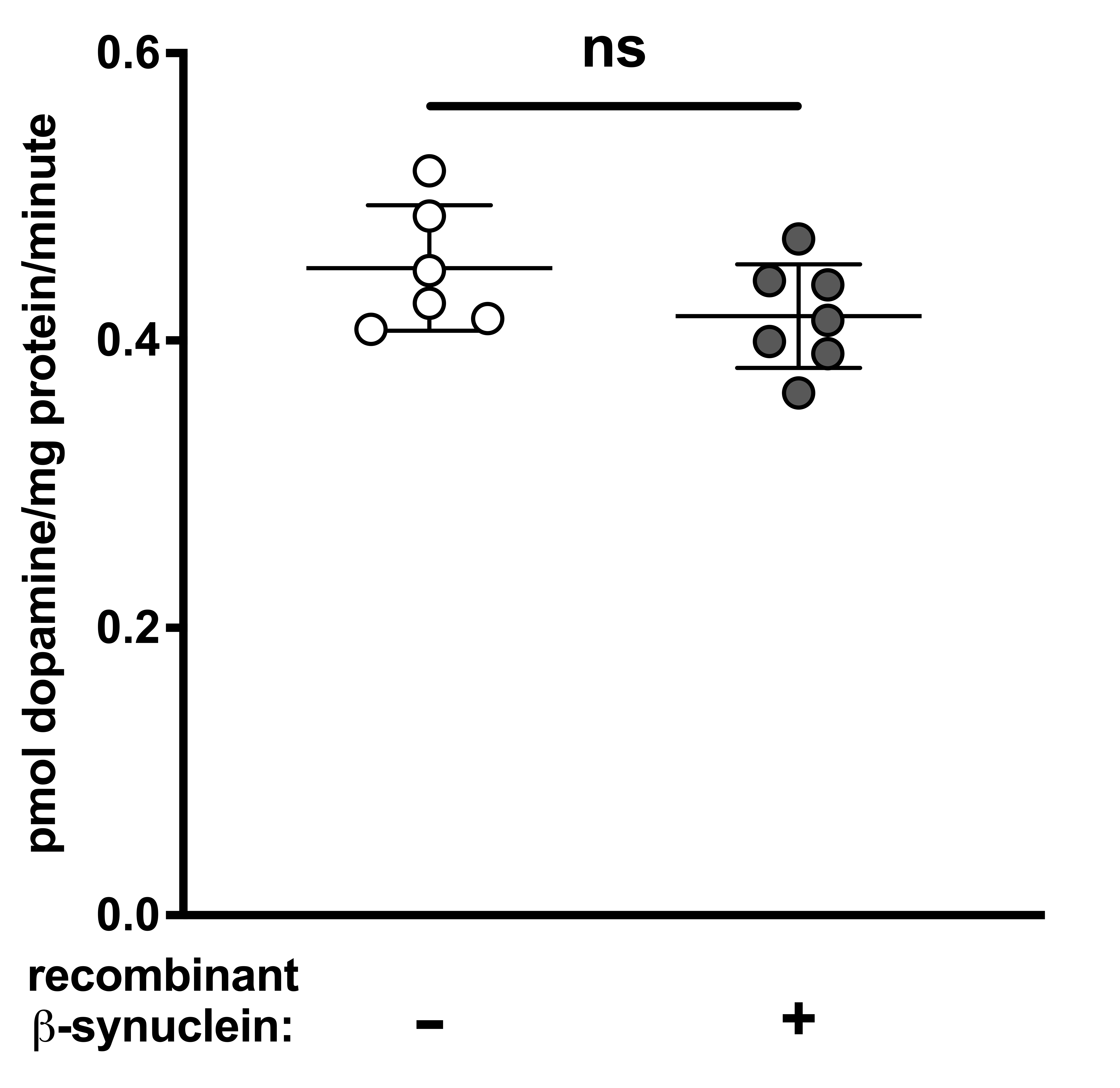


**Fig. S4. Recombinant β-synuclein does not affect dopamine uptake when added to already purified synaptic vesicles.**

Scatter plot shows means±SD of dopamine uptake by synaptic vesicles isolated from TKO mice in the presence of purified recombinant synucleins added to the uptake reaction mixture to final concentration of 20 µg/ml (n=7 for each condition from two independent experiments, ns, p=0.1807, Mann-Whitney U-test).

S3
